# Effect of beta-based sterilization on P(VDF-TrFE-CFE) terpolymer for medical applications

**DOI:** 10.1038/s41598-020-65893-2

**Published:** 2020-05-29

**Authors:** Nellie Della Schiava, Francesco Pedroli, Kritsadi Thetpraphi, Annalisa Flocchini, Minh-Quyen Le, Patrick Lermusiaux, Jean-Fabien Capsal, Pierre-Jean Cottinet

**Affiliations:** 1 Univ Lyon, INSA-Lyon, LGEF, EA682, F-69621 Villeurbanne, France; 2 Groupement Hospitalier Edouard Herriot, 69003 Lyon, France; 3 Université Claude Bernard Lyon 1 (Univ Lyon), 8 Avenue Rockefeller Lyon, F-69621 Villeurbanne, France

**Keywords:** Polymers, Biomaterials, Materials for devices

## Abstract

Electroactive polymers (EAP) are one of the latest generations of flexible actuators, enabling new approaches to propulsion and maneuverability. Among them, poly(vinylidene fluoride-trifluoroethylene-chlorofluoroethylene/chlorotrifluoroethylene), abbreviated terpolymer, with its multifunctional sensing and actuating abilities as well as its impressive electrostrictive behavior, especially when being doped with an plasticizer, has been demonstrated to be a good candidate for the development of low-cost flexible guidewire tip for endovascular surgery. To minimize the possibility of bacterial, fungal, or viral disease transmission, all medical instruments (especially components made from polymers) must be sterilized before introduction into the patient. Gamma/beta (γ/β) irradiation is considered to be one of the most efficient techniques for targeted reduction of microbials and viruses under low temperature, often without drastic alterations in device properties. However, radiation may cause some physical and chemical changes in polymers. A compromise is required to ensure sufficient radiation for microbial deactivation but minimal radiation to retain the material’s properties. The main idea of this study aims at assessing the electromechanical performances and thermal/dielectric properties of β-irradiated terpolymer-based sterilization treatment. Ionizing β-rays did not cause any significant risk to the neat/plasticized terpolymers, confirming the reliability of such electrostrictive materials for medical device development.

## Introduction

Endovascular surgery is an innovative, less invasive procedure that is used to treat problems affecting the blood vessels. An alternative to open surgery, endovascular surgery offers many advantages including a shorter recovery period, less discomfort, local or regional anesthesia, smaller incisions, less stress on the heart, and fewer risks for patients. Thus, endovascular techniques become most of time the first line treatment in vascular surgery even for very complex procedures^1–3^. These methods have currently propelled the growth and technical development of numerous intravascular guides and catheters to navigate different targeted arteries. Electroactive polymers (EAPs) have recently attracted a great deal of researcher interest because of their fascinating properties and high potential in medical application, particularly for active catheters or guidewire designs^4–7^. In previous studies, we demonstrated the feasibility of P(VDF-TrFE-CFE/CTFE) terpolymer to create a new generation of intravascular steerable guidewires^8,9^. Several advantages of the developed polymers are easy preparation, simple process, and high bending angle under relatively low input voltages, which makes them suitable materials for future generations of active guidewires. The proposed material likely allows some of the current technological issues relating to the development and control of the steerable guidewire to be overcome, such as the high cost, complex integration of micro-motor, and delayed response. The actuator device used in previous research contains di(2-ethylhexyl)phthalate (DEHP) plasticizer, which is not certificated as biocompatible for surgical procedures. To meet this medical requirement, diisononyl phthalate (DINP) plasticizer was chosen instead for this study because of its biocompatibility and safety for the patients and because of its exceptional ability to significantly enhance the electromechanical performances of the terpolymer^10^.

Medical devices today are multifunctional and are manufactured from a very wide variety of materials. Despite their frequently complex geometrical structures, each part has to be sterile. Particularly for invasive medical tools, sterilization becomes mandatory to avoid infections in patients. There are several methods of sterilization for endovascular surgery devices, and irradiation with γ/β rays and autoclaving with steam are the most frequently used methods. For autoclave sterilization, the necessary temperature is high, approximately 120 °C to 135 °C, which is not compatible with the terpolymer because it has a melting point of around 120 °C. Conversely, radiation-based sterilization is a cold method with no heat dependence and treatment can be efficient even at ambient temperature or sub-zero temperatures. Thus, this technique is compatible with many polymers that are sensitive to high temperature such as pharmaceuticals and biological samples. Moreover, γ/β radiation offers numerous advantages over traditional heat-based sterilization. In reality, only a single variable relating to the exposure dose/time must be monitored, making radiation sterilization simple and easy to control. Another benefit stems from the flexibility of this technique, which can sterilize any material with variable density, size, or thickness, and homogeneous or heterogeneous systems, regardless of temperature and pressure conditions.

Both β- and γ-rays are suitable for medical sterilization. The main difference between them lies in their depth of penetration and their dose rates. β-ray limited penetration is given at a high dose rate where a sterilization dose can be given within a few seconds or minutes. However, with γ-rays, deep penetration is traded for a low dose rate that may take several hours or even days to accumulate at a high dose. As in our study, all samples are very thin (i.e. few hundred of micrometers), and the use of β-radiation seems to be more suitable based on the best compromise between penetration and time consumption. Although radiation sterilization technologies have been extensively developed for medical applications, their effects on polymer characteristics have been poorly studied or published^11^. Irradiation destroys microorganisms and also changes material properties. These changes often depend on the radiation dose. Following radiation sterilization, application-specific tests should be carefully investigated to confirm which polymer is optimally suited to an individual application. Yang *et al*. studied the effects of e-beam ionizing radiation based γ-rays on P(VDF-TrFE-CFE) terpolymer and P(VDF-TrFE) copolymer^12^. To the best of our knowledge, a similar investigation dedicated to β-based sterilization has not been reported. The main objective of this study involves evaluating the β-radiation effect on the morphology and electromechanical properties of the terpolymer to determine if sterilization of our proposed material is possible for medical use.

## Methods

This section first describes the fabrication process of the P(VDF-TrFE-CFE) terpolymer that is doped with a plasticizer. Second, the characterization method including Differential Scan Calorimetry (DSC) measurements as well as electrical, mechanical, and electromechanical properties of the developed composites are then detailed.

### Polymer processing for neat and modified polymer

Polymeric films are realized via the solution casting method. The first step consists of dissolving the terpolymer P(VDF-TrFE-CFE) powder in 2-butanone solvent (also known as methyl-ethyl-ketone) at 25%wt. Monomeric composition of terpolymer is 62.2% for VDF, 29.4% for TrFE, and 8.4% for CFE expressed as molar fraction. The polymeric dissolution prepared in this manner is then casted on glass plate using a calibrated casting-knife to obtain polymer film with a thickness of approximately 55 µm ± 1 µm. Subsequently, casted films are heated up at 70 °C for 1 h to ensure complete solvent removal. Films are then peeled off and placed on polytetrafluoroethylene (PTFE) foils, and thermal annealing at 102 °C is performed to promote crystallinity^13^. The annealing temperature was chosen as the onset of the melting peak for the second melting of terpolymer powder that was determined via DSC.

To further improve the electromechanical performances of electrostrictive P(VDF-TrFE-CFE) terpolymer, modified polymeric films are realized by blending P(VDF-TrFE-CFE) terpolymer with 10%wt of a phthalate plasticizer^14^, which was performed at the first step during terpolymer dissolution. The addition of DINP plasticizer was chosen because of its biocompatibility as well as its ability to substantially enhance electromechanical coupling of the terpolymer^10^. The pure and modified EAPs were then subjected to high-energy β-irradiation at 50 kGy, which is a standard sterilization dose for medical instruments (according to industrial process of IONISOS). A comparison between the pristine and the sterilized samples in terms of thermal/electrical/electromechanical performance will be investigated in section III.

### Morphological characterization

To determine any possible morphological changes in the crystalline structure induced by the radiative sterilization treatment, DSC and X-ray diffraction (XRD) measurements were performed on all post-annealed and post-sterilized films. DSC, characterization of crystalline phase was done in terms of crystal content, average crystallite size and crystallite size distribution. XRD, allowing to structural characterizations at smaller scales, was also investigated.

Differential Scan Calorimetric measurements (DSCs) were performed on a SETARAM DSC131 EVO calorimeter. The thermal characterization aimed at determining potential effects on crystal morphology induced by the two different annealing treatments mentioned previously. The post-annealed polymeric films were heated from room temperature to 180 °C at a heating rate of 10 K/min. Crystallinity degree (or *Χ*_*c*_) is then calculated as the integral of the melting peak, which represents the melting enthalpy of the whole crystalline phases within the sample, divided by the melting enthalpy of a hypothetic 100% crystalline terpolymer. According to the work of R.J. Klein *et al*.^15^, the melting enthalpy value of the 100% crystallized terpolymer was estimated equal to 42 J/g, approximately. The second melting DSC ramp (10 K/min) that was performed on as-received terpolymer powder was implemented to identify the optimal annealing temperature.

The XRD allowed to check the crystal-phase quality by comparing positions and width at half maximum (FWHM) of the characteristic peaks so as to confirm a possible change in crystalline structure. In this study, the XRD was performed via an X’Pert Pro MPD Panalytical diffractometer using Cu-Kα radiation (λ = 1.5406 Å) of 45 kV and an electrical input of 40 mA in tandem with an incident-beam monochromator (Inc. beam Johansson 1xGe111 Cu/Co) and a X’Celerator detector. The diffraction patterns were recorded over an angular range of 10°–30° (2θ) where the characteristic peak relative to P(VDF-TrFE-CTFE) crystalline phase was localized^16^. A step length of the angular (2θ) equaled 0.017° with a counting time of 120 s/step. The extraction of the peak positions for indexing was performed via the X’Pert High Score.

### Electrical characterization

Broadband dielectric spectroscopy (BDS) was performed using a Solartron 1260 impedance-analyzer (AMETEK Advanced Measurement Technology, England). Ambient temperature dielectric spectra are acquired under an alternative voltage of 1V_peak-peak_ amplitude at a frequency range of 10^−1^–10^6^ Hz.

The dielectric strength of the selected materials was determined by applying a DC voltage linear ramp at a slew rate of 500 V/s. For each polymeric film, 10 to 16 breakdown events were recorded to show the probability distribution of electrical failures.

### Electromechanical performances

To efficiently assess the electromechanical actuation of the sterilized samples via beta radiation, a dedicated set-up based cantilever structure was built^17^. First, 50 × 10-mm² terpolymer film specimens of 55 µm thickness were coated on both sides using 20-nm-thick gold electrodes. Second, using an adhesive energy transfer tape, the electroded terpolymer was attached on a 100-µm-PET inactive substrate. Finally, the three-layer cantilever was laminated at room temperature under high vacuum for 15 min to cure the adhesion of each layer. The multilayer cantilever was clamped on one end, while the other end was free and its deflection (unimorph tip displacement) was measured using a laser sensor (Baumer CH8501). A full description of the experimental setup was detailed in our previous work^18^.

As reported in literature^19,20^ and in our previous works^10,13^, terpolymer P(VDF-TrFE-CFE) commonly exhibits a purely electrostrictive behavior. Indeed, the mechanical strain response shows a quadric dependence with the applied electric field, and as a result, its direction is not dependent on the polarity of the voltage excitation. Furthermore, with respect to ferroelectric polymers, this class of EAPs offer the technological advantage of no poling steps (or polarization process) required before their use. Accordingly, cantilever samples are excited by unipolar sinusoidal electric field with frequency of 0.1 Hz.

One of the most relevant parameters characterizing the electromechanical performance of the transversal strain *S*_31_ can be deduced from the deflection measurements of a unimorph under quasi-static conditions, according to the following expression^21^:

1 $${\delta }_{0}=\frac{3{L}^{2}}{2e}\frac{2AB{(1+B)}^{2}}{{A}^{2}{B}^{4}+2AB(2+3B+2{B}^{2})+1}{S}_{31}$$

where *δ*_0_ is the cantilevered tip deflection, *A* = *Y*_*substrate*_*/Y*_*poly*_ and *B* = *e*_*substrate*_*/e*_*poly*_; *Y*_*substrate*_ and *Y*_*poly*_ respectively depict the Young modulus of the substrate (*Y*_*substrate*_ = 3.24 GPa) and of the polymer (*Y*_*poly*_ = 150 MPa for pure terpolymer and 50 MPa for plasticized one); *e*_*substrate*_, *e*_*poly*_ respectively depict the substrate thickness (*e*_*substrate*_ = 100 µm), the polymer thickness (*e*_*poly*_ = 55 µm); *L* and *e* are the length (i.e. 50 mm) and the total thickness of the cantilever (i.e. *e*_*substrate*_ + *e*_*poly*_ = 155 µm), respectively.

The model based on Eq. (1) is only reliable for relatively small deflections. Consequently, all materials explored in this study will be excited under electric fields where the tip deflection of the unimorph does not exceed 3 mm (corresponding to a strain less than 1%). Finally, simultaneously to cantilever tip deflection, EAP output current is recorded and polarization loops are calculated.

## Results and Discussion

### Morphological analysis

**Figure 1:**
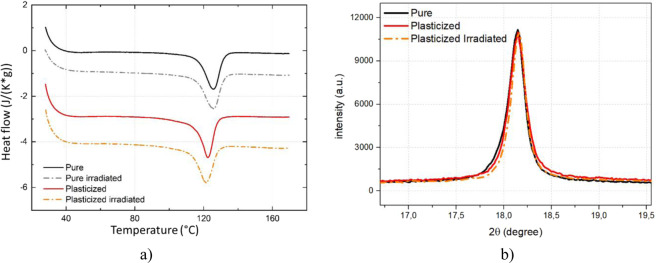
Morphological analysis of the pristine and irradiated neat and plasticized terpolymers: (**a**) DSC thermographs, and (**b**) Diffraction analysis (the result of the irradiated pure sample is not shown as it is identical to the non-irradiated pure one).

Figure 1a shows the DSC thermal analysis results of the pure and 10% DINP plasticized terpolymers with/without the β-irradiation process. Similar to our previous work^18^, the plasticized terpolymer slightly decreased the melting peak temperature (i.e. 122.3 °C) compared to the pure terpolymer (i.e. 125.8 °C). However, the melting peak enthalpy was slightly higher, increasing from 18.6 J/g for the neat sample to 20.1 J/g for the modified sample. This phenomenon is probably caused by increased molecular chain mobility, which facilitate the polymer crystallization especially when being doped with a plasticizing substance^18^.

To better understand the influence of β-radiation on EAP morphology, analysis of the melting peak together with the thermographs in Fig. 1 are summarized in Table 1, which contains complete information about quality and distribution size of crystal domains. The pertinent parameters are comprising the melting temperature (T_melting_), the melting enthalpy variation (ΔH_melting_), the full width at half maximum (FWHM) and the crystallinity degree (or χ_c_). While T_melting_ and ΔH_melting_ refer respecctivelly to the quality and quantity of crystalline phase, FWHM gives indication regarding the crystalline size distribution^22,23^. Similar results were obtained for both with- and without-sterilized samples, confirming that β-based sterilization does not alter the morphology of the crystalline phase or the thermal behavior of the fabricated EAPs.

**Table 1:** DSC thermograph analysis results.

	T_melting_ (°C)	ΔH_melting_ (J/g)	FWHM (°C)	χ_c_ (%)
Pure	125.8	18.6	9.4	44.3
Pure irradiated	126.2	18.8	10.0	44.7
Modified	122.3	20.1	8.3	47.8
Modified irradiated	121.8	19.7	9.3	46.9

A deeper investigation of crystal structure was carried out based the XRD analysis. The diffraction spectra (Fig. 1b) shows diffraction peaks perfectly superimposable for either irradiated or non-irradiated sample because there was no variation in peak position (*2θ* = 18.16°) or peak width (FWHM = 0.17°). Therefore, no modification in crystal lattice space was found confirming that the β-based sterilization did not alter the crystalline phase conformation. Similar to the DSC thermographs, the XRD diffraction peaks do not exhibit any variation in crystal size distribution for all samples.

### Dielectric spectroscopy

In this subsection, broadband dielectric spectroscopy in different frequencies and temperature was investigated to further assess the β-radiation effect on the dielectric behavior of the pure and plasticized terpolymers. Indeed, the range extended in temperature and frequency used in this study makes it possible to obtain information relating to the physical mechanism behind the changes in the permittivity according to these parameters.

**Figure 2:**
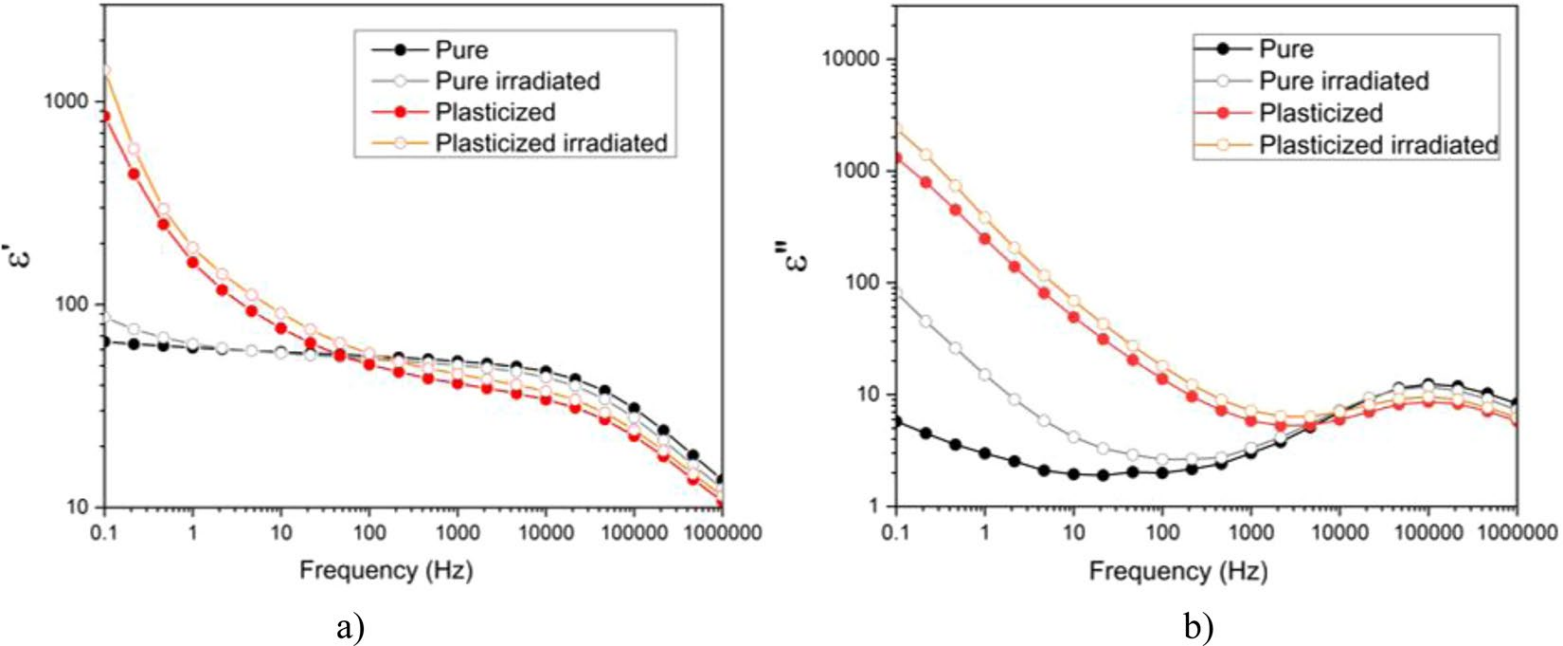
BDS spectra for the pristine and irradiated material: (**a**) real part of relative permittivity, and (**b**) imaginary part of relative permittivity.

Figure 2a illustrates the real part of relative permittivity of the four samples over a wide frequency range of 10^−1^–10^6^ Hz. Under high frequencies (i.e. greater than 1 kHz), the pure materials (i.e. black and gray curves) showed the typical dielectric behavior of a polar polymer above its glass transition temperature. In other words, thanks to large mobility of polymer molecules, the contribution of molecular dipole polarization (or orientational polarization) is highly frequency dependent, and as a result, the total relative permittivity^10,12^ increases from 13 to 52, within this frequency range (i.e. from 1 MHz down to 1 kHz). Whereas at low frequencies, the dielectric permittivity remains almost constant^24^.

For the modified terpolymer (red and orange curves), its permittivity values considerably increased at low frequencies because of boosted interfacial phenomena that typically occur for this material structure^23^. The interfacial polarization is largely improved by ion conduction and molecular mobility in plasticized samples thanks to the augmented free volume in the polymer amorphous phase^25–27^. This effect can be seen in Fig. 2b where under low frequencies, the related dielectric losses of the modified EAP are definitively more significant than the neat sample, regardless of the radiative procedure.

Figure 2b also shows that the radiative treatments gives rise to dramatically increase permittivity imaginary part (ɛ″) at a very low frequency for the pure terpolymer, increasing from 4 (before sterilizing) to 70 (after sterilizing). Such a huge increase in dielectric losses of the neat material may stem from the higher mobility of the intrinsic molecular chains after being treated with β-radiation, resulting in expanding inter-chain distance of the polymer’s internal chemical crosslinks. As widely studied in literature^28,29^ for typical EAP like PVDF (i.e. P(VDF-TrFE) copolymers, high-energy radiation results in expanding the inter-chain distance within the polymer amorphous phase due to chemical crosslinks among polymer chains. Such an irradiated polymer matrix makes ionic impurities easier conduct through the sample, leading to increase permittivity imaginary part (ɛ″) at low frequency (Fig. 2b).

This phenomenon, however, is not the same as the plasticized EAP where the dielectric losses of both irradiated and pristine samples are almost the same, having negligible discrepancies under the whole frequency range. Because the plasticized terpolymer demonstrated extremely high molecular mobility^30,31^, some ruptured molecular chains from β-radiation processing probably only slightly influenced the polymer structure^32,33^.

In conclusion, these results demonstrated that the β-radiation led to a variation in dielectric performances of the neat P(VDF-TrFE-CFE) terpolymer while there was no major change in the plasticized sample. To some extent, the sterilization procedure may affect the morphological modification of EAP, specifically favoring its interfacial polarization related to the molecular mobility^34,35^.

**Figure 3:**
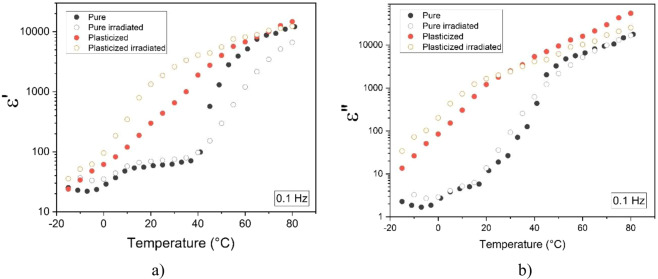
Temperature dependence of dielectric properties for the pristine and irradiated neat and plasticized terpolymers at 0.1 Hz: (**a**) real part of relative permittivity, and (**b**) imaginary part of relative permittivity.

As reported on Cheng *et al*.^36^, for high-energy-electron irradiated P(VDF-TrFE), the temperature dependence of the dielectric properties changes a lot. In our case for the electrostrictive terpolymer, whose characteristics is not the same to the ferroelectric polymer as P(VDF-TrFE) copolymer, the dielectric responses of both pure and plasticized terpolymers, especially the dielectric losses, are demonstrated to be almost independent to the irradiation sterilization at a wide temperature range from –15 °C to 80 °C. The maximum temperature chosen in this test is limited to 80 °C, which is closed to the glass transition temperature of the terpolymer. Figure 3 illustrated temperature dependence of dielectric properties for all the four samples under a dynamic excitation of 0.1 Hz. Such a low frequency is chosen in order to meet application in electromechnical actuation that will be described in subsection III.4. As it can be seen in Fig. 3a, there exists somewhat difference in permittivity constant when the pure and the plasticized samples are radiatelly treated. For the dielectric losses (cf. Fig. 3b), on the other hand, turns out to be similar between the sterilized samples and the untreated ones, confirming no significant temperature change of the proposed material when being subjected by high energy *β*-irradiation.

It is interesting to note that for all the samples, large variation of permittivity constant (ε′) and dielectric losses (ε″) in term of temperature has been recorded. Particularly, under low frequency and high temperature, the loss factor increases sharply, which corresponds to typical of a DC conductivity mechanism. In conclusion, the results in Figs. 2 and 3 revealed that, for EAPs in general, frequency and temperature are the two key parameters having high impact on dielectric properties and as a result, can strongly affect to the polarizability of material. In terpolymer, the molecular orientation is technically random with its semi-crystalline structure and therefore, its dielectric behavior strongly depends on chain mobility and charge motion. Consequently, it is difficult to achieve electrostrictive polymer whose permittivity as well as dielectric losses are stable over wide range of frequency and/or temperature. A definition of operating frequency and temperature dedicated to specific applications is a crucial step for advanced design of actuator materials. In our study, the device is supposed to be performed under room temperature or inside human’s body where temperature variation is relatively small, from 18 °C to 37 °C. Furthermore, low dynamic of actuator is required during surgical procedure, i.e. less than a few tenths of Hz. Based on these specifications, it is possible to be assumed that our developed material can maintain its dielectric performance at such temperature and frequency range.

### Electrical breakdown

Electrical breakdown strength (*E*_*BD*_) is theoretically estimated based on the following power law relation^37^:

1 $${E}_{BD}=k\,{d}^{-n}$$

where *d* is the polymer thickness, *k* and *n* are constants depending on material properties like dielectric behavior, homogeneity, geometry, etc.

The decrease in dielectric strength as a function of thickness is can be explained by the volume effect. Actually, the probability to encounter defects in the polymer exponentially increases with the volume. Therefore, enhancing the dielectric thickness between the electrodes leads to statistically increase number of defects occurring in the active volume. Another reason is due to the fact that, thinner film allows to facilitate heat evacuation within the material when this one is excited by a high voltage level, resulting in significantly decrease breakdown probability.

Regarding Eq. (1), the relative uncertainty of the breakdown electric field ($\Delta {E}_{BD}/{E}_{BD}$) can be estimated as:

2 $$\frac{\Delta {E}_{BD}}{{E}_{BD}}=n\frac{\Delta d}{d}$$

where $\Delta d/d$ is the relative uncertainty of the film’s thickness (i.e. ≈2% for all samples).

**Figure 4:**
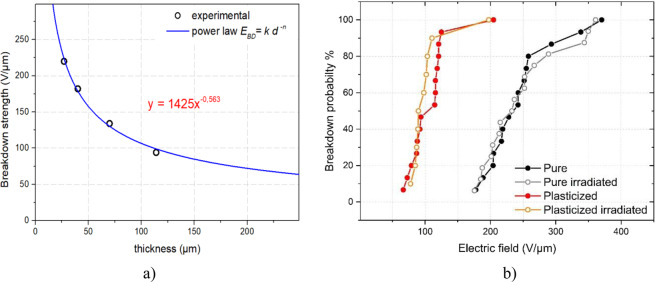
(**a**) Breakdown strength versus thickness of P(VDF-TrFE-CTFE) terpolymer films. (**b**) Experimental DC breakdown electric field distribution, lines are drawn to guide the eye.

To determine the parameter *n*, which is relating to the speed of decay, empirical breakdown strength of terpolymer films versus their thicknesses was carried out in order to fit with the model of Eq. (1). As expected in Fig. 4a, experimental result is consistent to the theoretical model of Eq. 1, confirming high influence of sample’s thickness to electrical breakdown. The fitting parameter *n* is found equal to 0.56, which is in agreement to those published on the literatures^38–40^. Similar approach was applied to the plasticized terpolymer whose value of *n* was obtained nearly 0.65. Through improvement of polymer fabrication process, it is possible to reduce the value of parameter *n* by structural defects and enclosed impurities, giving raise to drastically enhanced electrical breakdown.

Finally, the uncertainty of 2% (corresponding to 1 µm) in thickness leads to variation of approximately 1% to 1.2% in breakdown electric field (*E*_*BD*_), respectively for the pure and modified samples in either irradiated or non-irradiated case. Measured values of breakdown electric field (*E*_*BD*_) together with their uncertainties were shown in Table 2. It can be noticed that the variation of breakdown electric field is relatively small, i.e. less than 2 V/µm for the modified terpolymer and 3 V/µm for the pure one, regardless to the irradiation process.

**Table 2:** DC breakdown electric field from the Weibull analysis.

	E_BD*_*63_ (V/µm)	k
Pure	267 ± 3	4.7
Pure irradiated	271 ± 3	4.4
Modified	119 ± 2	3.3
Modified irradiated	116 ± 2	3.1

Figure 4b illustrates the experimental breakdown probability *P*(*E*) versus electric field for the four different polymeric films (neat/modified and with/without β-radiation). Each dot represents one experimental breakdown measurement. As a result, the breakdown strength is estimated according to the following Weibull model:

3 $$P(E)=1-exp\left[-{\left(\frac{E}{{E}_{BD\_63}}\right)}^{k}\right]$$

where *P(E)* is the breakdown probability at a given electric field *E*, *E*_*BD_*63_ is the parameter corresponding to the breakdown electric field for a failure probability of 63%, and *k* is the shape factor defining the distribution spread^41^. These parameters of the four samples determined from the Weibull model are summarized in Table 2.

The results show that for a given failure probability, the EAP filled with DINP plasticizer had a lower electrical breakdown strength, e.g. 119 ± 5 V/µm for the modified terpolymer compared to 276 ± 5 V/µm (2-fold greater) for the pure terpolymer. Because of some defects in the modified terpolymer, adding an excessive amount of plasticizer can limit the operating voltage, which will strongly affect to the strain production. However, in our application where a low electric field was used (i.e. less than 50 V/µm), the breakdown probability for the terpolymers is extremely low, even for those filled with 10% DINP plasticizer content. The electrical strength of either a pure or modified sample was completely conserved after a β-radiation treatment.

### Electromechanical performance

To evaluate the electromechanical performances of the pristine and irradiated materials, actuation characterization based on tip displacement measurements was performed using unimorph cantilevers, as described in the previous section II.4.

**Figure 5:**
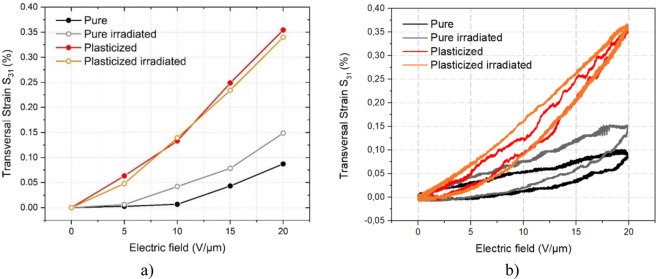
(**a**) Transversal strain amplitude of pure and plasticized terpolymers with/without β-irradiation treatment for different applied electric fields, lines are drawn to guide the eye. (**b**) Electromechnical hysteresis of the four samples under a given applied voltage of 20 V/µm amplitude.

Figure 5a displayed the maximum experimental transversal strain *S*_31_ for the pure and plasticized EAPs before and after sterilization for different applied voltages. The radiative treatment did not alter the electromechanical activity of the plasticized terpolymers. As also confirmed by the above dielectric spectroscopy, β-radiation could not further expand the already large molecular mobility of the polymer amorphous phase, resulting in actuation behavior that was unchanged. However, the transversal strain *S*_31_ of the pure terpolymer exhibits a superior electromechanical response for the sterilized sample. The enhanced electromechanical conversion for the irradiated pure terpolymer can be justified by its higher dielectric permittivity, as shown in Fig. 2.

Figure 5b depicts experimental transversal strain *S*_31_ of all the four samples that was powered by a unipolar sinusoidal waveform of 20 V/µm amplitude. As expected, their strain-versus-electric-filed response displays a hysteresis behavior, which reflects the electromechanical losses generated by the sample during actuation. Such a hysteric characteristics attests a phase shift between the mechanical response and the applied input voltage. Interestingly, the extension of the electromechanical hysteresis turns out to be similar for all the four samples, regardless either the addition of plasticizing agent or the irradiation treatment. This result is in contrast to the one shown in Fig. 2b where the modified terpolymer exhibits higher dielectric losses than the pure one.

It has been previously shown that in the case of dielectric polymers, the electrostrictive strain under electric field can be mainly attributed to Maxwell forces induced by dipolar orientation within the material^42^. In the longitudinal direction, the compressive Maxwell strain and the mechanical energy density under electric field are given by^43^:

4 $$S=\left(\frac{{\varepsilon }_{0}{\varepsilon }_{r}}{Y}\right){E}^{2}$$

In our case where the applied electric field is relatively low (i.e. 20 V/µm), the induced mechanical strain observed in pure or plasticized samples originates from the Maxwell stress effect. As a result, the resulting strain only depends on the squared electric field, by assuming that the dielectric permittivity is constant for such a low range of electric field. The theoretical model of Eq. (4) successfully reflects the empirical results obtained in Fig. 5, where a quadratic behavior between the strain response and the applied electric field has been achieved.

**Figure 6:**
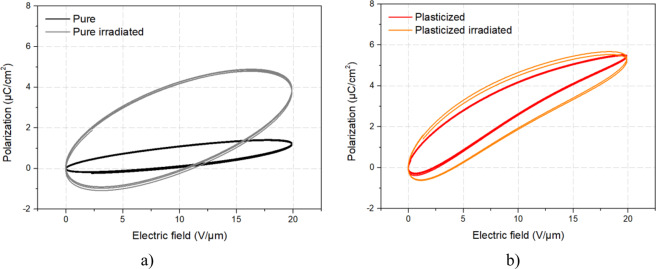
Polarization loops for (**a**) pure terpolymers with/without irradiation, and (**b**) plasticized terpolymers with/without irradiation.

Figure 6a,b describes the polarization loops of the four samples obtained during their actuation. The result confirms hysteresis behavior which, this time, reflects electrical losses of EAPs when being driven under significant voltage (i.e. 20 V/µm). As observed, the pure irradiated terpolymer shows much larger hysteresis with respect to all other samples, particularly compared to the untreated pure sample. Plasticized samples (Fig. 6b), on the other hand, outstandingly display a pretty narrow polarization loops despite their important losses of ionic conductivity based on dielectric spectroscopy of Fig. 2b. This phenomenon principally caused by higher molecular mobility of plasticized terpolymer as opposed to the pure material^10^, leading to fast saturation of the ionic conductivity, and therefore increase dielectric losses under low frequencies^13,44,45^. In conclusion, higher intrinsic dielectric losses of the plasticized terpolymer does not have too much impact on electromechanical and electrical losses of the material during actuation.

**Figure 7:**
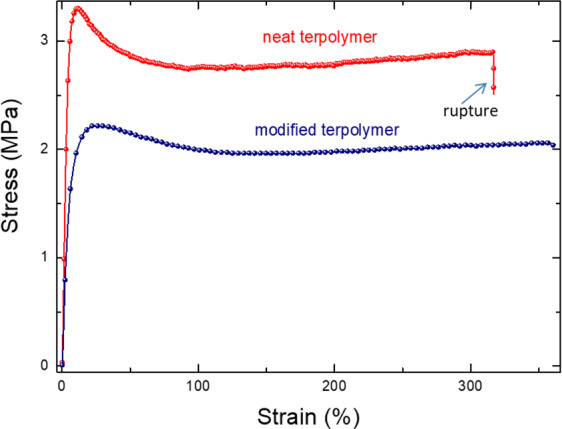
Longitudinal strain versus stress for different electrostrictive composites.

To better assess the high performance of the plasticized polymer, the mechanical properties characterizing the material rupture based on the stress/strain behavior is described in Fig. 7. It can be seen that the modified sample gives rise to a higher critical strain level, which is in agreement with the fact that the Young modulus of the neat terpolymer is superior over the modified one. As expected for both samples, at the beginning, the stress linearly increases with the strain and then a saturation of the stress occurs when the strain becomes more important (>10%). In this study, we only focus on the linear elastic domain of the polymer whose Young modulus can be deduced equal to approximately 30 MPa for the plasticized terpolymer and 100 MPa for the pure one. It is noteworthy that the sterilization process does not modify the mechanical properties of the pure and the plasticized terpolymers. This result is in consistent to the electric-field-induced mechanical strain (Fig. 5) where no important change has been detected between the irradiated samples and the untreated samples.

## Example of Application

There is an increasing trend toward replacing the conventional methods of open surgery with minimally invasive (MIS) procedures called robotic surgery. This novel technique gives the surgeon new capabilities for manipulating surgical instruments with more complex procedures while enhancing precision. Furthermore, it offer patients several benefits like small incision, less trauma, minimal scarring, and faster recovery time. Advances in new MIS method strongly depend on development of new materials that must be biocompatible and able to satisfy new biomedical requirements. In our recent work, we have shown high potential of soft electroactive polymer for cardiovascular application in steering smart guidewire^8^. The result demonstrated a possibility of controlling the miniaturized actuator inside arteries and/or veins, thanks to high bending angle of the flexible material that was driven by very low applied voltage.

The 3D smart guidewire is performed by extruding an all-organic EAP to design a form of tube. For building a mixture between terpolymer and plasticizer, solution casting method^30^ is carried out to create thin film that can be easily cut into small pellets of 5 × 5 mm². The extrusion process starts by feeding the pure or modified terpolymer from a hopper into the barrel of a Laboratory Mixing Extruder (LME, Dynisco, Inc.). The material is gradually melted using the mechanical energy generated by turning screws at a rotational speed of 180 RPM and by heaters arranged along the barrel of around 150 °C. The molten material is then forced into a die of 130 °C, which shapes the polymer into a long tube that hardens during cooling. After that, the tube was cut into the desired length and recrystallized in an oven at the onset of the melting peak determined by a Setaram EVO 131 Dynamic Scanning Calorimetry (DSC). To create electrical connection, a center electrode is made from conducive Carbon grease that is properly filled up the tube. Two identical outer electrodes with semicircular shape are sputtered on the surface of a cylindrical EAP, allowing the smart guidewire to bend in both directions^8,9^. Two gaps separating these two electrode is drawn to avoid short-circuit. The detail dimension of the whole smart guidewire is resumed in Table 3.

**Table 3:** Dimension of the smart guidewire.

Description	Value
Thickness of guidewire	0.2 mm
Length of guidewire	60 mm
Diameter of the central electrode	1 mm
Larger of each outer electrode	1.7 mm
Thickness of each outer electrode	20 nm
Gap between both electrode 1 and 2	0.5 mm

It is noteworthy that the fact of introducing a conducive grease inside the guidewire is not well adapted to medical environment. The next step of our future work consists of using co-extrusion technique that allows to extrude EAP and continuously filled with a conductive material in order to form a single terpolymer tube together with active electrodes.

**Figure 8:**
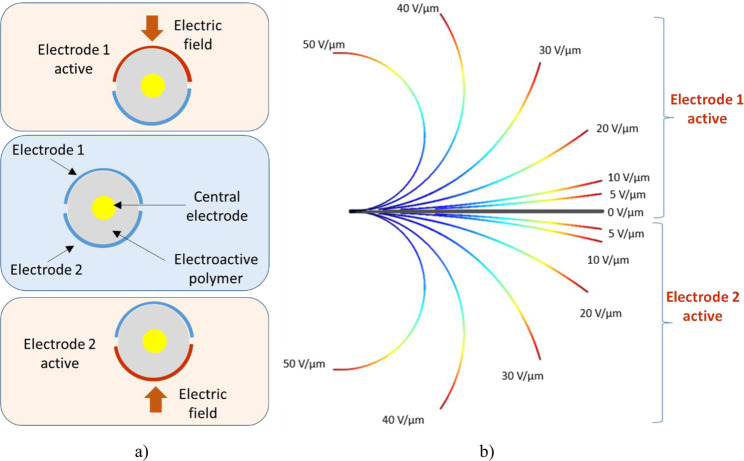
(**a**) Cross-section view of the smart guidewire-based EAP driven by an electric field alternatively applied on 2 electrodes; (**b**) Electromechanical deformation of the irradiated plasticized terpolymer under different electric field excitation.

**Figure 9:**
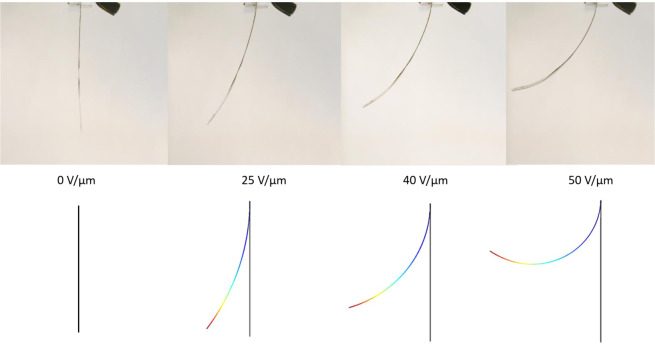
Experimental results of the smart guidewire obtained using the plasticized and irradiated prototype.

Figure 8a illustrates the working principle of the developed 3D smart guidewire. The architecture of the steerable guidewire is composed of a conductive electric wire surrounded by a two degrees of freedom (2-DOF) electrostrictive terpolymer. Indeed, by alternatively activating electrodes 1 and 2, it is possible to easily control the movement of the EAP. Figure 8b shows the modeled electromechanical behavior-based Comsol finite element method for a 10%wt DINP plasticized terpolymer under different levels of input voltage excitation. Figure 9 revealed that the experimental results are in agreement with the theoretical model where the discrepancy is less than 10%. The displacement response of the proposed actuator has been practically demonstrated to be sufficient to lift a rigid guidewire tip. Further empirical tests in the *in vitro* or *in vivo* conditions should be investigated in future work to better assess the new device’s performance.

## Conclusion

This work confirmed a reliability of our novel material that was based on 10% DINP plasticized terpolymer for a new generation ultra-flexible active bending system that can be used as a steerable guidewire tip in endovascular surgery. Sterilization of all medical devices is required to reduce the contamination risk for the patient. Because of extensive investigations into the bactericidal effects of radiation and continuing improvements in irradiation technology, ionizing radiation is increasingly being used in this field. However, radiation-based sterilization is an extremely sensitive, critical step because biomedical devices should maintain their chemical and physical properties through different stages of processing including terminal sterilization. Our study revealed that the ionizing β-ray did not significantly alter the intrinsic properties of the neat/plasticized terpolymers, causing no change in its electromechanical activities or to its thermal and electrical characteristics. These results suggest the possibility of using EAP as biocompatible and sterilizable material that can be implemented in future smart guidewires for endovascular surgery.
